# Precise Deposition of Polydopamine on Cancer Cell Membrane as Artificial Receptor for Targeted Drug Delivery

**DOI:** 10.1016/j.isci.2020.101750

**Published:** 2020-11-01

**Authors:** Hoda Safari Yazd, Yu Yang, Long Li, Lu Yang, Xiaowei Li, Xiaoshu Pan, Zhuo Chen, Jianhui Jiang, Cheng Cui, Weihong Tan

**Affiliations:** 1 Department of Chemistry, University of Florida, Gainesville, FL 32611-7200, USA; 2 Molecular Science and Biomedicine Laboratory (MBL), State Key Laboratory for Chemo/BioSensing and Chemometrics, College of Chemistry and Chemical Engineering, College of Life Sciences, and Aptamer Engineering Center of Hunan Province, Hunan University, Changsha 410082, China; 3 Institute of Molecular Medicine (IMM), Renji Hospital, Shanghai Jiao Tong University School of Medicine, and College of Chemistry and Chemical Engineering, Shanghai Jiao Tong University, Shanghai 200240, China

**Keywords:** Drug Delivery System, Cancer, Biomaterials

## Abstract

Compared with conventional chemotherapy and radiotherapy, targeted molecular therapy, e.g., antibody-drug conjugates or aptamer-drug conjugates, can specifically identify overexpressed natural receptors on the cancer cell, perform targeted release of anticancer drugs, and achieve targeted killing of tumor cells. However, many natural receptors are also expressed on non-cancer cells, thereby diverting the targeting molecules to healthy cells. By generating artificial cell surface receptors specific to diseased cells, aptamer-drug conjugates can identify these artificial receptors, improve therapeutic efficacy, and decrease the minimum effective dosage. In this study, we use high K^+^ and high H_2_O_2_ of the tumor microenvironment (TME) to produce polydopamine only on living cancer cell membrane. Owing to the significant reactivity of polydopamine with amino groups, e.g., the amino group of proteins, polydopamine can deposit on tumor cells and act as “artificial receptors” for targeted delivery of anticancer drugs with amino groups, in other words, amino-containing drugs and protein drugs.

## Highlights

• Polydopamine (PDA) generation catalyzed using G-quadruplex DNAzyme
• TME high K^+^ and H_2_O_2_ employed to produce PDA only on cancerous cells membrane
• PDA generated and deposited on cancerous cells and acted as artificial receptors
• PDA artificial receptors facilitated targeted delivery of drugs with amino groups

## Graphical Abstract

**Figure:**
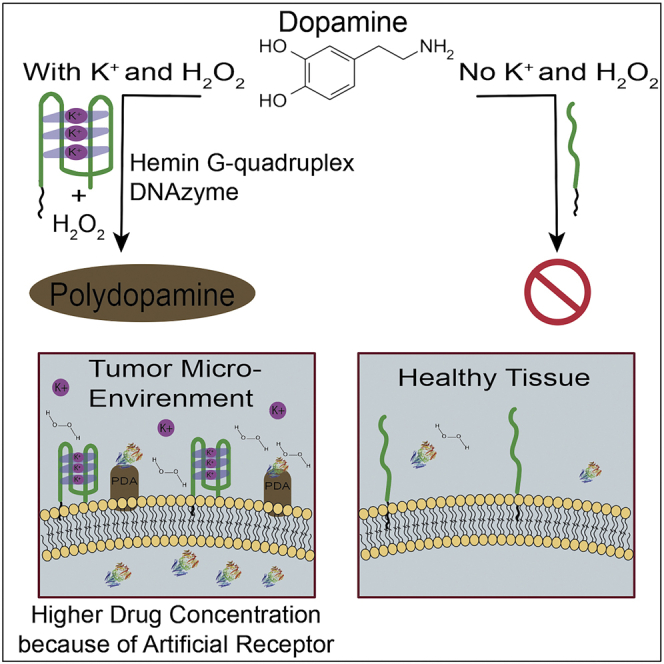

## Introduction

Current cancer therapy, such as anticancer chemotherapeutic drugs or radiation therapy, mostly cannot differentiate healthy from cancer cells owing to a lack of selectivity (Yang et al., 2016). Targeted drug delivery systems, on the other hand, promise to extend the therapeutic impact of drugs by enhancing delivery to the targeted tissue, increasing target/nontarget tissue ratio, avoiding nonspecific toxicity in healthy tissues, and reducing side effects. Targeted drug delivery can reduce drug dosage and the accompanying drug toxicity, as well as enhance therapeutic efficiency at equivalent plasma concentrations (Chari, 2008; Yang et al., 2018). To this end, several molecular targeting elements such as nucleic acid scaffolds, antibodies, and small organic molecules with recognition ability to diseased cells have been extensively investigated. Most recently, aptamers, which are single-stranded nucleic acid ligands selected by Systematic Evolution of Ligands by Exponential Enrichment (SELEX), have been developed as a novel family of molecules that rival antibodies in both diagnostic and therapeutic applications (Zhu et al., 2015; Xuan et al., 2018; Li et al., 2019). Aptamers are highly specific with a broad range of ligands, such as cells, proteins, and small molecules, and have been used as a molecular targeting element for incorporation into aptamer-drug conjugates (ApDCs) for targeted therapy. Because they can provide specificity at the molecular level, aptamers can be used to enhance the efficacy of experimental and/or commercial drugs in clinical applications (Tuerk and Gold, 1990; Fang and Tan, 2010; Sefah et al., 2010; Kamaly et al., 2012). Generally, tumor cells show a specific pattern of overexpressed membrane-associated proteins, particularly receptors. Many natural receptors are known to be significantly overexpressed in various cancer tissues and contribute to assembling nutrients for uncontrolled cancer growth and survival, tumor initiation, and stimulus for angiogenesis (Goel and Mercurio, 2013; Feigin et al., 2014). Traditional targeted therapy based on targeting molecules, including aptamers and antibodies, can recognize and directly bind to some specific overexpressed natural receptors on the diseased cell membrane and lead to release of anticancer drugs, achieving targeted killing of tumor cells with fewer side effects (Akhtar et al., 2014). However, many natural receptors associated with cancer are also expressed on noncancer cells, albeit at a lower level of expression, reducing the efficacy of targeted delivery and causing damage to healthy cells. The generation of artificial cell surface receptors, instead of natural receptors, offers the promise of increased specificity. In other words, artificial receptors could act as mimics drawing one hundred percent of targeted drug delivery to the cell of interest. Artificial receptors could be various types of molecules, such as proteins, polymers, or synthetic organic compounds, and could be used to promote efficient cellular uptake (Niikura et al., 2011), generate an artificial transmembrane signal transduction mechanism (Langton et al., 2017a, 2017b), and efficiently deliver therapeutics and probes (Hussey and Peterson, 2002; Boonyarattanakalin et al., 2004; Peterson, 2005; Sun et al., 2008; Hymel and Peterson, 2012). Artificial receptors can be produced on the cancer cell surface by using cancer cell characteristics and the tumor microenvironment (TME). Polydopamine (PDA) with low toxicity, excellent biocompatibility, and ultrastability (Liu et al., 2013; Han et al., 2016; Poinard et al., 2018) is widely known for its specific reactivity to the amino groups through Michael addition and Schiff base reaction (Lee et al., 2009; Liu et al., 2014; Li et al., 2017; Sileika et al., 2011). Thus, PDA with amino groups can be considered as a safe and efficient artificial receptor for anticancer drugs in the TME. In the last few years, TME, characterized by such features as ECM, exosome, or acidity, have been extensively employed as targets for targeted therapy (Joyce, 2005; Pickup et al., 2014; Paolicchi et al., 2016; Roma-Rodrigues et al., 2017; Yu et al., 2017; Iessi et al., 2018; Capello et al., 2019). The unique characteristics of the TME have a considerable impact on cancer progression, development, invasion, and metastasis (Hanahan and Coussens, 2012; Quail and Joyce, 2013; Joyce and Fearon, 2015; Spill et al., 2016). One such feature is the concentration of potassium ions in the TME, which is 5–10 times higher (40 mM) than that of healthy tissues (5 mM) (Eil et al., 2016). Hydrogen peroxide (H_2_O_2_) is also present in the TME in significant concentration (Szatrowski and Nathan, 1991).

In this study, we produce polydopamine (PDA) on the tumor cell membrane surface as an artificial receptor through catalysis of dopamine using G-quadruplex DNAzyme, which only shows high catalytic activity in environments with high concentration of K^+^ and H_2_O_2_. Briefly, we employed AS1411 (Yang et al., 2016), a guanine (G)-rich DNA oligonucleotide, and hemin (iron (III)-protoporphyrin IX) to form a hemin/G-quadruplex complex to quickly catalyze dopamine to polydopamine. Interestingly, this can only happen in the TME, as the concentration of extracellular potassium ion is significantly higher than that of healthy cells, in turn resulting in the generation of stabilized hemin-G quadruplex DNAzyme, which cannot be achieved in healthy tissues. It is fortuitous that utilization of stabilized DNAzyme for its peroxidase activity can also be achieved through H_2_O_2_ molecules abundantly available in the TME. This means that hemin-G quadruplex DNAzyme can be stabilized and exhibit (HRP)-like activity, but only in the tumor microenvironment, and we exploited this remarkable feature to generate PDA on tumor tissues. Because PDA can specifically react with amino groups, it can deposit on the cancer cell membrane and act as an artificial receptor, facilitating specific targeted delivery of anticancer drugs with amino-like cisplatin and protein drugs such as saporin.

## Results

### Hemin-G Quadruplex DNAzyme Catalyzes Polydopamine Polymerization

**Scheme 1:**
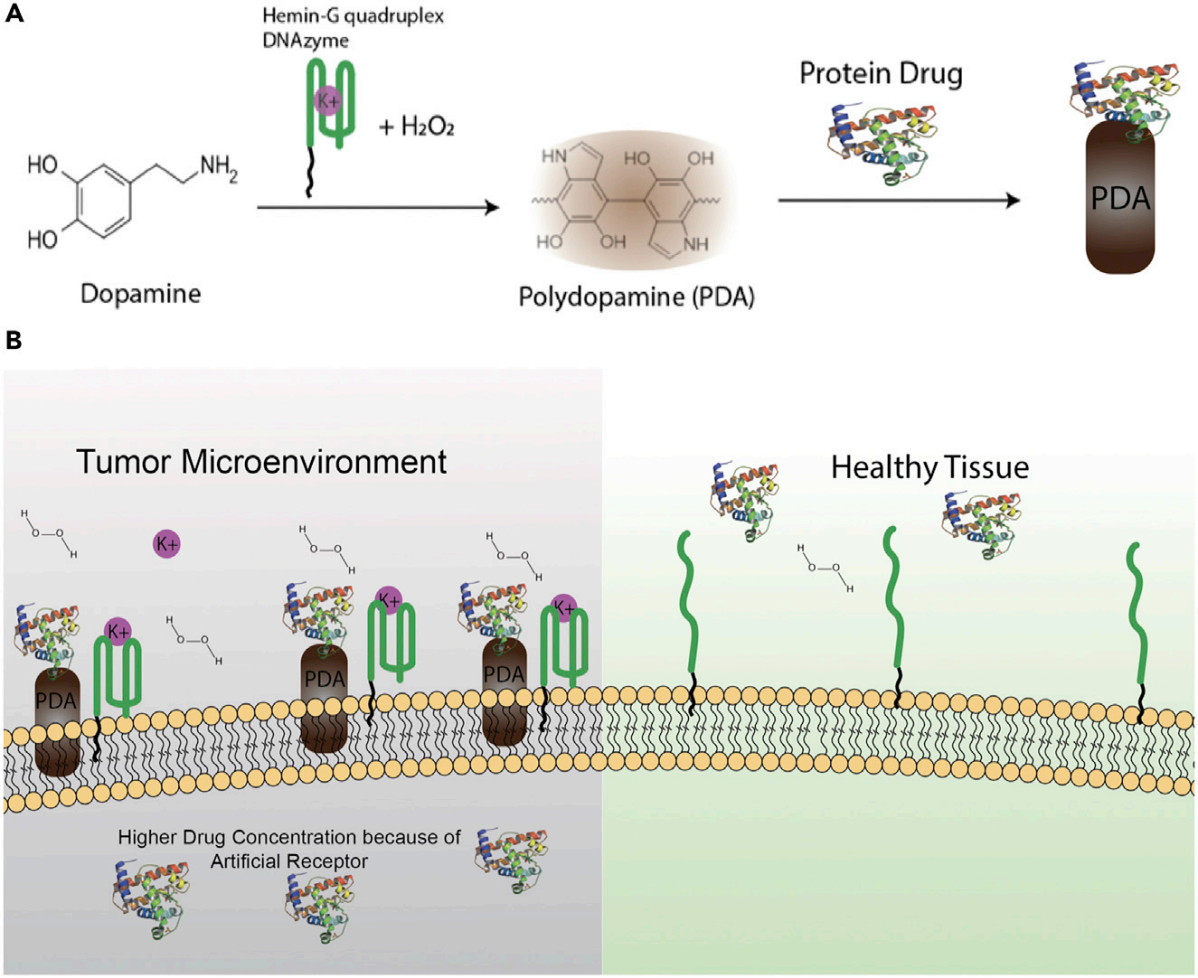
Schematic Representation of Polydopamine Polymerization Mechanism (A) PDA artificial receptors can be catalyzed using G-quadruplex DNAzyme and specific characteristics of TME. (B) PDA can only deposit on the cancerous cell membrane and act as artificial receptors to deliver protein drugs and amino-containing small drugs to them.

In order to engineer the hemin-G quadruplex DNAzyme constructs, lipid-AS1411 (see detailed sequences in Supplemental Information Table S1) with G-rich sequence was synthesized to form stable G-quadruplex in the presence of potassium ions. Next, potassium ion solution with final concentration of 40 mM was added into 2 μM AS1411 solution to stabilize G quadruplex. Afterward, hemin was added into this mixture. Hemin binds with high affinity to G quadruplex, and after incubating the mixture at room temperature for 50 min, hemin-G quadruplex DNAzyme was produced. At this point, the created DNAzyme structure could exhibit predominant peroxidase-like activity and accelerate the production of PDA in the presence of hydrogen peroxide. Therefore, hydrogen peroxide (H_2_O_2_) with a final concentration of 500 μM was added to the constructed hemin-G quadruplex DNAzyme, followed by the addition of dopamine hydrochloride solution to the mixture. As illustrated in Scheme 1A, by generating G-quadruplex DNAzyme on the cell membrane and employing some specific characteristics of TME, we can accelerate the polymerization of dopamine to polydopamine specific to the cancer cell membrane as artificial receptors. PDA is widely known for its exceptional reactivity to amine groups; therefore, drugs with amino groups can react and attach to polydopamine. As shown in Scheme 1B, as a result of high extracellular concentration of K^+^ and H_2_O_2_ in the TME, PDA was only deposited on the membrane of cancer cells to then act as artificial receptors to deliver protein drugs and amino-containing small drugs with selectivity. This means that no PDA can be deposited on healthy cell membrane.

**Figure 1:**
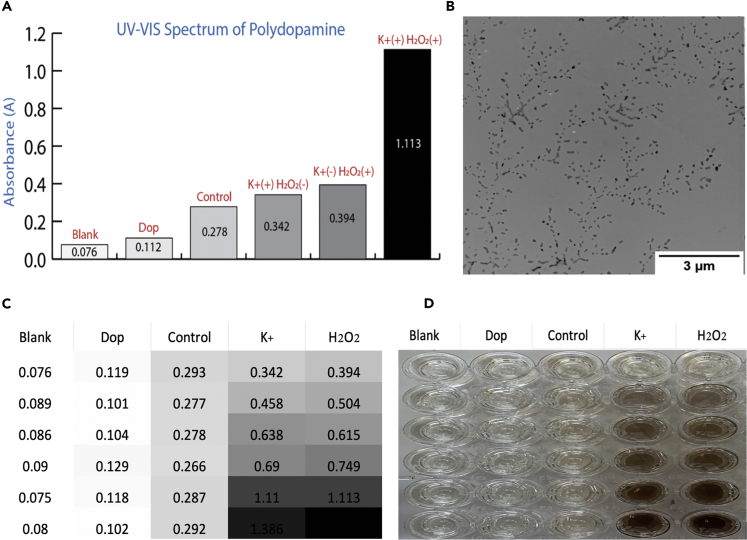
PDA Polymerized by Our Method (A) UV-VIS absorption spectra of produced polydopamine. Group Blank: water; Group Dop: dopamine only; Group Control: dopamine and hemin-lipid-AS1411 without K^+^ and H_2_O_2;_ Group K^+^ (+), H_2_O_2_ (−): control only in the presence of K^+^; Group K+ (−), H_2_O_2_ (+): control only in the presence of H_2_O_2_; Group K^+^ (+), H_2_O_2_ (+): control in the presence of K^+^ and H_2_O_2_. (B) TEM image of PD nanoparticles. (C) Heatmap of UV-VIS absorption of polydopamine in various concentration of K^+^ and H_2_O_2_: Blank, Dop, and Control same as (A); K^+^: gradient concentration of potassium ion, high to low: 0 mM, 10 mM, 20 mM, 30 mM, 40 mM, and 50 mM with constant concentration of H_2_O_2_ = 500 μM; H_2_O_2_: gradient concentration of hydrogen peroxide, high to low: 0 μM, 50 μM, 100 μM, 200 μM, 500 μM, and 1,000 μM with constant concentration of K^+^ = 40 mM. (D) Corresponding photograph of C, showing the color change.

We first studied the catalytic ability of G quadruplex DNAzyme for PDA production. Because PDA can rapidly deposit on proteins, BSA, as the PDA deposition anchor point, was added to the mixture. Next, the produced PDA was measured by UV-VIS-NIR at 450 nm. As shown in Figure 1A, PDA is conventionally produced from oxidative polymerization of dopamine in a basic aqueous medium. However, this method is considerably slow, and it takes a comparatively long time to produce a small amount of polydopamine. However, potassium ions play a key role in the construction of G-quadruplex DNAzyme, and hydrogen peroxide (H_2_O_2_) has an important role in rapidly catalyzing the production of PDA. In the presence of both hydrogen peroxide and potassium ions, a brown-black polydopamine can be produced after only 30 min following the addition of dopamine hydrochloride, indicating that hemin-G quadruplex DNAzyme can utilize the hydrogen peroxide to catalyze the polymerization of PDA. TEM imaging shows the production of polydopamine nanoparticles, and their rapid generation and deposition on BSA are shown in Figure 1B (Figures S1 and S2). In Figure 1C, the production of PDA was tested for the existence of various concentration of K^+^ (from 0 mM to 50mM) and H_2_O_2_ (from 0 μM to 500 μM) and the catalytic activity of DNAzyme in different conditions. As shown in the heatmap, by increasing the concentration of potassium ion and hydrogen peroxide, a higher amount of PDA was produced. Meanwhile, the corresponding photograph of produced PDA based on Figure 1C showed that the amount of produced polydopamine is directly correlated with the concentration of potassium ions and hydrogen peroxide (Figure S3, pH effect on PDA production).

### Polydopamine Generation and Deposition on the Cancerous Cell Membrane

**Figure 2:**
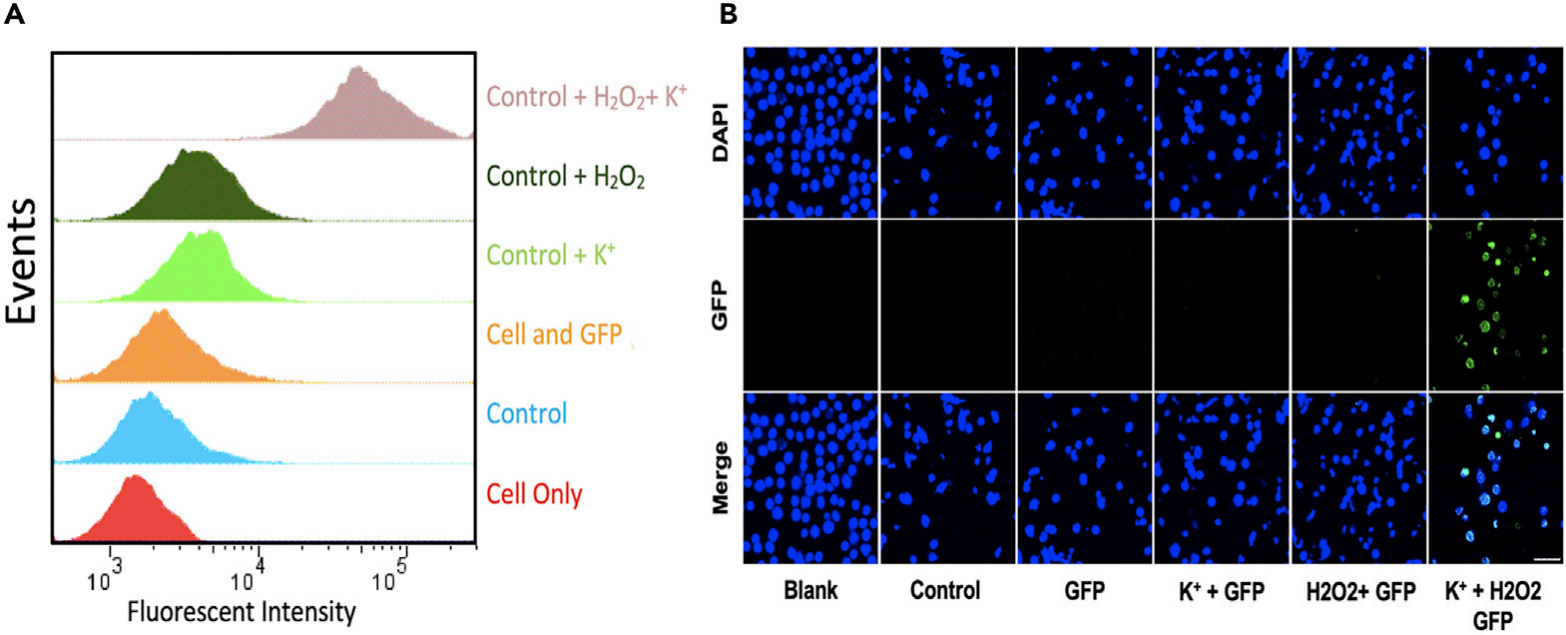
PDA Produced and Deposited on Cell Membrane (A) Flow cytometry histogram of CCRF-CEM cells treated with HEPES buffer (cell only group), Chol-AS1411, hemin, and EGFP (“control” group), only enhanced green fluorescent protein (“GFP” group), control + K^+^ (“K^+^ + GFP” group), control + H_2_O_2_ (“H_2_O_2_+ GFP” group), and control + K^+^ + H_2_O_2_ (“H_2_O_2_+ K^+^ + GFP” group). Results show that EGFP stained the deposited PDA on the cell membrane but only in the presence of K^+^ and H_2_O_2_. EGFP concentration = 500 nM. (B) Confocal microscopy images of CCRF-CEM cells treated with different reagents to produce PDA; EGFP concentration = 2 μM; scale bar: 50 μm.

Encouraged by the precise deposition of PDA on BSA using hemin-G quadruplex DNAzyme, we further investigated the possibility of using PDA polymerization with the proposed method on cell membrane. To accomplish this, we employed cholesterol-AS1411 conjugates to anchor on the cell membrane (see Transparent Methods section within the Supplemental Information for further details). Because of hydrophobic interaction between cellular phospholipid layer and cholesterol, cholesterol-DNA conjugates could insert in cell membrane. We chose CCRF-CEM cells as a model cell line and added potassium ion (40 mM) and hydrogen peroxide (500 μM) in cell culture media to mimic the TMEs with a high concentration of hydrogen peroxide and potassium ion. First, cholesterol-AS1411 conjugates were added to the cells and incubated for 40 min at 4°C; then a final concentration of 40 mM potassium ion solution was added and incubated for another 40 min at 4°C. For the next step, hemin was added to the mixture and stabilized DNAzyme was successfully generated. Finally, PDA was produced on the cell membrane after H_2_O_2_ and dopamine addition. In the presence of H_2_O_2_ and K^+^, dopamine converts to polydopamine quickly such that it can be localized and deposited on the cell membrane proteins (Figure S4). To evaluate whether the produced PDA on the cell membrane could be considered as an efficient artificial receptor, enhanced green fluorescent protein (EGFP) and FITC-7T-NH2 were chosen as model proteins. EGFP is a protein that exhibits bright green fluorescence and could be used to study the dynamic changes of cellular processes in living cells. Here, EGFP with high amine density, as the model protein, could be attached to deposited PDA on the cell membrane, generating a fluorescence signal with which to evaluate PDA production. Therefore, EGFP was added after the completion of PDA production in the cells, and then flow cytometry was employed to quantify and compare EGFP delivery efficiency. As shown in Figure 2A, an obvious shift in flow cytometry histogram occurs when the concentration of potassium ion and hydrogen peroxide is high enough to produce PDA on the cell membrane, acting as artificial receptors for our model protein and showing available PDA on the cell membrane by the amount of EGFP for attachment, as indicated by the uppermost peak in the histogram. For all other flow cytometry experiments, no apparent shift gives evidence of the amount of EGFP attached to the cell membrane, suggesting that only negligible amount resulted from the small amount of accessible PDA, which showed much lower fluorescence on the cells. Then, under the same conditions, a lower concentration of EGFP (100 nM) was applied, in turn leading to a lower amount of attached EGFP to the cell membrane and a smaller shift in flow cytometry histogram (Figure S5). Still, a distinct shift and fluorescence signal could be seen in the abundance of K^+^ and H_2_O_2_. After using EGFP as a model protein, an amino-containing fluorescence molecule was also used to perform as a small-molecule model, and it could recognize PDA production on the cell membrane. FITC-7T-NH2 is a short oligonucleotide sequence, with fluorescein isothiocyanate (FITC) directly coupled onto the 5′-end and amino modifier coupled onto the 3′-end. The amino group in the sequence could attach to the produced PDA on the cell membrane, and a coupled FITC fluorescent molecule is on the other side. A fluorescence signal on the cell membrane can be detected by using flow cytometry. Accordingly, flow cytometry histograms confirmed that this model small-molecule could attach to cell membrane and generate fluorescence signal only in the presence of K^+^ and H_2_O_2_, which is consistent with the results of EGFP, indicating that PDA artificial receptors play a major role for targeted delivery of small molecules to the cells (Figure S6).

Next, confocal laser scanning microscopy (CLSM) imaging was used to monitor the targeting efficiency of PDA as an artificial receptor in the presence of K^+^ and H_2_O_2_. For this experiment, cells were fixed in 2% formalin solution for 7 min, stained with 4′,6-diamidino-2-phenylindole (DAPI) to localize their nucleus, and washed prior to confocal microscopy observation. Confocal laser scanning microscopy images confirmed that samples incubated with both H_2_O_2_ and K^+^ exhibited a strong green fluorescence signal on the cell membrane, as well as in the cytoplasm of CCRF-CEM cells, indicating that EGFP not only bound to target CCRF-CEM cells, but were also internalized (Figure 2B). Based on the results shown in Figure 2, produced PDA can be employed as artificial receptor for targeted delivery of protein drugs to the cells in the presence of both K^+^ and H_2_O_2_ (Cell uptake analysis Figure S7).

### Deposited Polydopamine on the Cancerous Cell Membrane Act as Artificial Receptors for Targeted Drug Delivery

**Figure 3:**
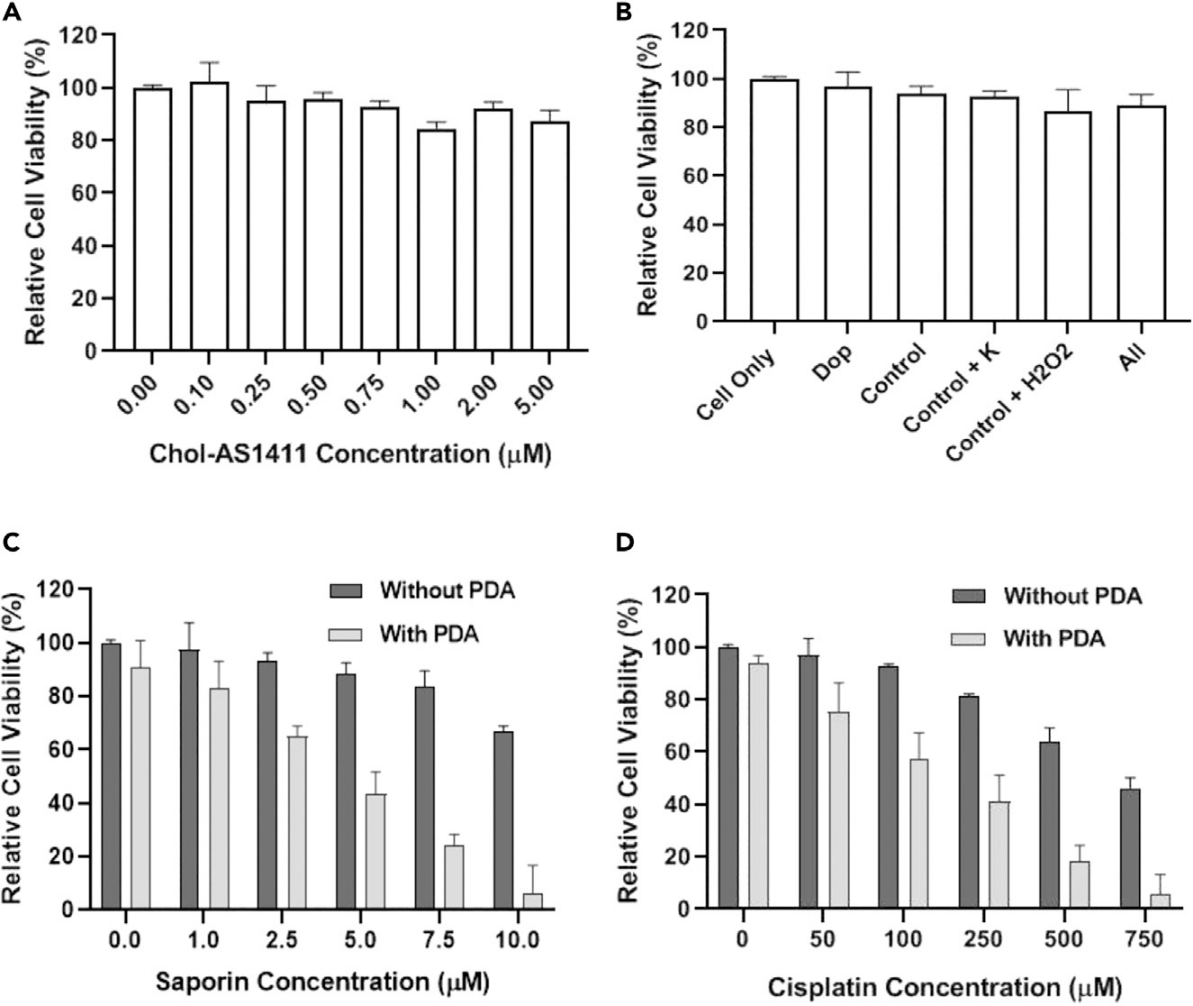
Generated PDA on the Cell Membrane Act as Artificial Receptors Cell viability of CCRF-CEM cells treated with various reagents followed by verification of cell viability by MTS assay. The error bars represent the standard deviation of three parallel experiments. (A) Cells treated with different concentration of Chol-AS1411. (B) Cells treated with PDA reagents, Group Dop: dopamine only; Group Control: dopamine and hemin-lipid-AS1411 without K^+^ and H_2_O_2_. (C) Cells treated with different concentration of saporin (with/without artificial receptor). The “Without PDA” labels contain all the compounds such as dopamine, and all the reaction conditions are kept precisely the same as the “With PDA” wells. (D) Cells treated with different concentration of cisplatin (with/without artificial receptor). The “Without PDA” labels in Figures 3C and 3D contain all the compounds such as dopamine and all the reaction conditions are kept exactly same with the “With PDA” wells.

Inspired by the well-defined delivery of FITC-7T-NH2 and EGFP by utilizing PDA, we eventually explored the capacity of this artificial receptor to perform functional targeted drug delivery in cancer therapeutics and precise therapy. For this purpose, cisplatin, an anticancer and cytotoxic chemotherapeutic small drug with an amino group, was selected. Then, saporin, an anticancer protein drug that impedes protein synthesis with the aid of ribosome inactivation, was used. Potent cytotoxicity of chol-AS1411 and PDA production was then examined by using the MTS assay (3-(4,5-dimethylthiazol-2-yl)-5-(3-carboxymethoxyphenyl)-2-(4-sulfophenyl)-2H-tetrazolium)). CCRF-CEM cells (30,000) were washed and incubated with the desired concentration of cholesterol-AS1411 (from 0 μM to 5 μM), and MTS assay was employed to measure cell viability. As shown in Figure 3A, chol-AS1411 was not toxic to CCRF-CEM cells, even at concentration up to 5mM. Cytotoxicity effect of PDA production was also examined, and based on the MTS results in Figure 3B, even the addition of all reagents did not cause dramatic cytotoxicity under our experimental conditions, indicating excellent biocompatibility and applicability for using this method in the cell's microenvironment. These results indicate that PDA production in TME does not have a toxic impact on cell viability; also, PDA's deposition on the cell membrane did not affect the cell viability and the membrane structure, as shown in Figure S4. In addition, in PDA degradation point of view, *in vivo* studies performed by Langer's group have confirmed that polydopamines were almost entirely degraded after eight weeks and suggested that polydopamine could be degraded *in vivo* in a timely manner (Bettinger et al., 2009).

**Figure 4:**
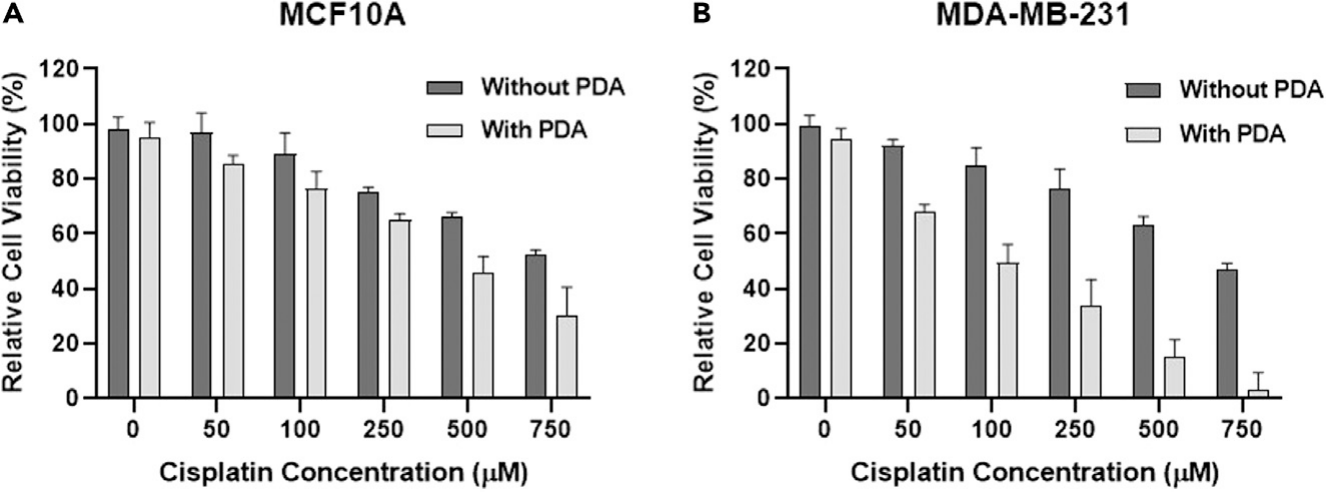
Generated PDA on the Cell Membrane Act as Artificial Receptors Cell viability of MCF10A and MDA-MB-231 cells treated with various concentration of cisplatin in the absence and presence of artificial receptors followed by verification of cell viability by MTS assay. The error bars represent the standard deviation of three parallel experiments. (A) MCF10A cells as the control and non-cancerous cells treated with different concentration of cisplatin (with/without artificial receptor). (B) MDA-MB-231 cells as the cancerous cells treated with different concentration of cisplatin (with/without artificial receptor). The “Without PDA” labels in Figures 3C and 3D contain all the compounds such as dopamine, and all the reaction conditions are kept exactly same with the “With PDA” wells.

To evaluate targeted therapy efficiency based on PDA artificial receptor, saporin (0–10 μM), as a model protein drug, and cisplatin (0–750 μM), as the amino-containing model small-molecule drug, were exposed to CCRF-CEM cells right after the production of PDA on their cell membrane and then incubated at 37°C in 5% CO_2_ for 6 h. Cells were then washed, and fresh RPMI-1640 complete medium was added to the cell's solution. Culture medium was allowed to grow at 37°C in 5% CO_2_ for 48 h, and, eventually, MTS assay was used for cytotoxicity determination. As demonstrated in Figures 3C and 3D, after saporin and cisplatin incubation, CCRF-CEM cells with the artificial receptor showed significant reduction of cell viability to 10%, whereas without generation of artificial receptor, no obvious cytotoxicity was observed for the CCRF-CEM cells (Figure S8, the cytotoxicity study also confirmed these results). Also, according to the results in Figures S9 and S10, the IC50 of cisplatin decreased from 1,082.68 μM to 225.16 μM with artificial receptors, and for saporin, it decreased from 34.73 μM to 3.469 μM. To further investigate the PDA deposition on the cancerous and non-cancerous cell membrane, if both are available in tumor micro-environment, MDA-MB-231, a cancerous breast cell line, and MCF10A, a non-cancerous breast cell line, were employed. PDA was generated and deposited on both cell lines, and cisplatin was applied as amine-containing cytotoxic drug as described in Supplemental Information, and finally cell viability was evaluated using MTS assay. As it is shown in Figure 4, the cisplatin cytotoxicity is obviously higher in MDA-MB-231 cell line in the same exact condition with MCF10A. These results indicate that the higher protein density in cancerous cell could help the PDA to specifically deposit on their cell membrane compared with non-cancerous cells. Significantly, PDA artificial receptors delivered cisplatin and saporin to inhibit cell proliferation with higher efficiency than observed for cells without artificial receptors at any drug concentration level, further confirming a superior drug delivery efficiency using artificial receptors in biological settings. Besides, these results show that if PDA artificial receptors are available on the cancerous cell membranes, there is a considerable probability that the amine-containing drug cargo will deposit on the cells as soon as it is added to the TME and kills the cells significantly faster than there are no artificial receptors on the cells. Thus, it may be concluded that PDA can be produced and serves as an artificial receptor on the cell membrane for precise targeted delivery of anticancer drugs with amino groups, but only in the presence of K^+^ and H_2_O_2_. Moreover, to determine the specificity of the designed PDA artificial receptors with amino-containing drug in the living subject, additional in-vivo studies are needed.

### Conclusion

In summary, we have successfully generated PDA as artificial receptors on the cancer cell membrane by employing specific TME features. Owing to the peroxidase-like activity of hemin-G quadruplex DNAzyme, polydopamine molecules were quickly produced and deposited on the cancer cell membrane to facilitate targeted delivery of anticancer drugs with amino groups. Taking advantage of the unique feature of cancer cells, their membrane and their microenvironment, only these artificial receptors were targeted by amino-containing drugs. By using saporin and cisplatin as two model drugs, we were able to realize much higher efficiency in inhibition of the cell proliferation in the presence of artificial receptors.

### Limitation of the Study

The result of this study indicates that deposited PDA artificial receptors on the cancerous cell membrane could facilitate the delivery of amino-containing drugs. Further *in vivo* studies are needed to determine the specificity of the designed PDA artificial receptors in the living subjects' complex environment and also to evaluate the physiological and biochemical characteristics of this system in order to ultimately develop potential therapeutic strategies using these artificial receptors.

### Resource Availability

#### Lead Contact

Further information and requests should be directed to and will be fulfilled by the Lead Contact, Weihong Tan (tan@hnu.edu.cn).

#### Materials Availability

This study did not generate new unique reagents.

#### Data and Code Availability

All data produced or analyzed for this study are included in the published article and its Supplemental Information files.

## Methods

All methods can be found in the accompanying Transparent Methods supplemental file.

## Supplemental Information

Document S1. Transparent Methods, Figures S1–S10, and Table S1

## Footnotes

Supplemental Information can be found online at https://doi.org/10.1016/j.isci.2020.101750.
