# Supplementary material for: Precise Deposition of Polydopamine on Cancer Cell Membrane as Artificial Receptor for Targeted Drug Delivery
Source: iScience. 2020 Nov 1;23(12):101750. doi: 10.1016/j.isci.2020.101750 (PMC7749375; doi:10.1016/j.isci.2020.101750)
Supplement: Document S1. Transparent Methods, Figures S1–S10, and Table S1 [file mmc1.pdf]

iScience, Volume *23*

Supplemental Information

Precise Deposition of Polydopamine on Cancer Cell Membrane as Artificial Receptor for Targeted Drug Delivery

Hoda Safari Yazd, Yu Yang, Long Li, Lu Yang, Xiaowei Li, Xiaoshu Pan, Zhuo Chen, Jianhui Jiang, Cheng Cui, and Weihong Tan

Supporting Information

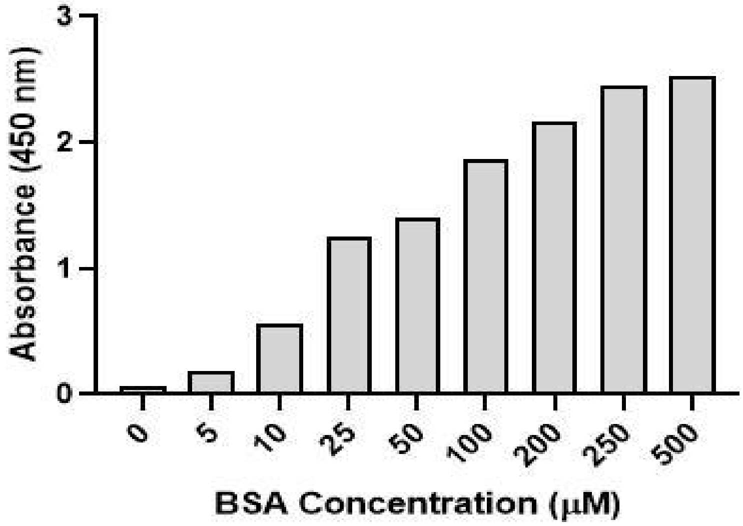

Figure S1. PDA in the presence of Bovine Serum Albumin (BSA), Related to Figure 1.

UV-VIS absorption spectra of the produced polydopamine (PDA) in the presence of different concentration of Bovine Serum Albumin (BSA).

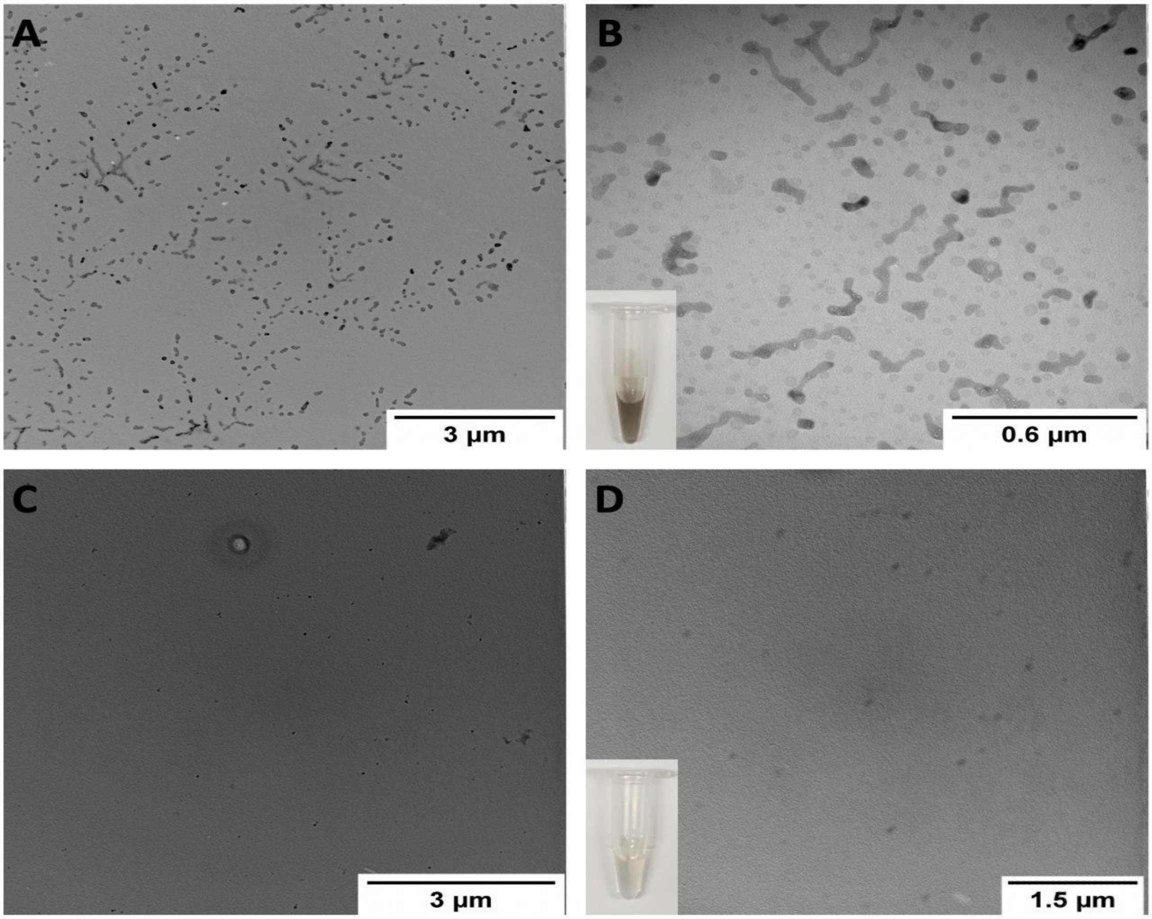

Figure S2. Transmission Electron Microscopy (TEM) image of PDA nanoparticles, Related to Figure 1.

A and B shows the TEM images of a mixture that contains BSA, which confirms that PDA molecules are confined near BSA. C and D shows the TEM images of a mixture that does not contain BSA and PDA molecules quickly diffuse away and did not generate dark spots.

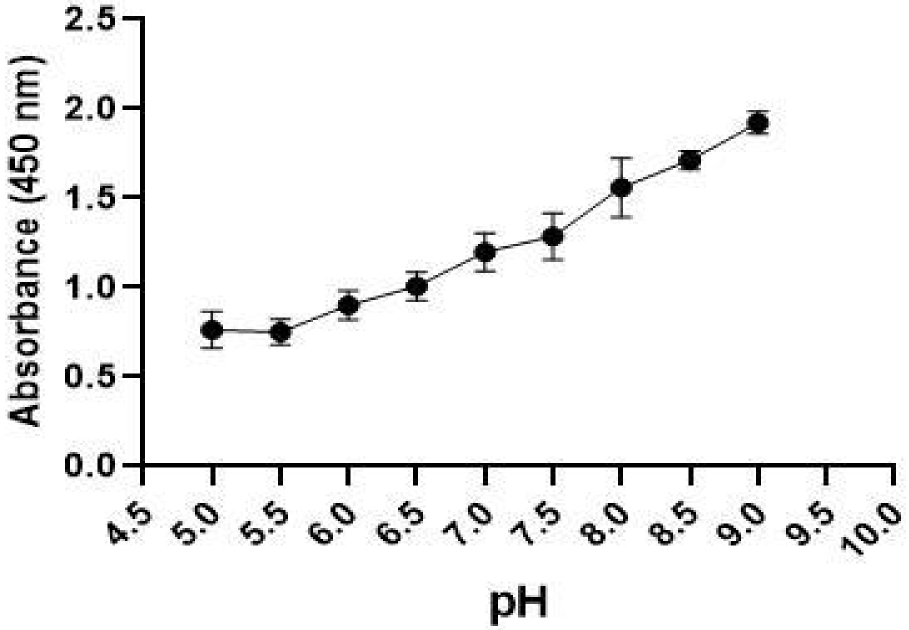

Figure S3. Absorbance of PDA solutions resulting from different initial pH values, Related to Figure 1.

To investigate the pH effect on the synthesis of PDA, we generated the PDA as it is described before and the initial pH adjusted to the required values by adding HCl and/or NaOH using a calibrated pH meter.

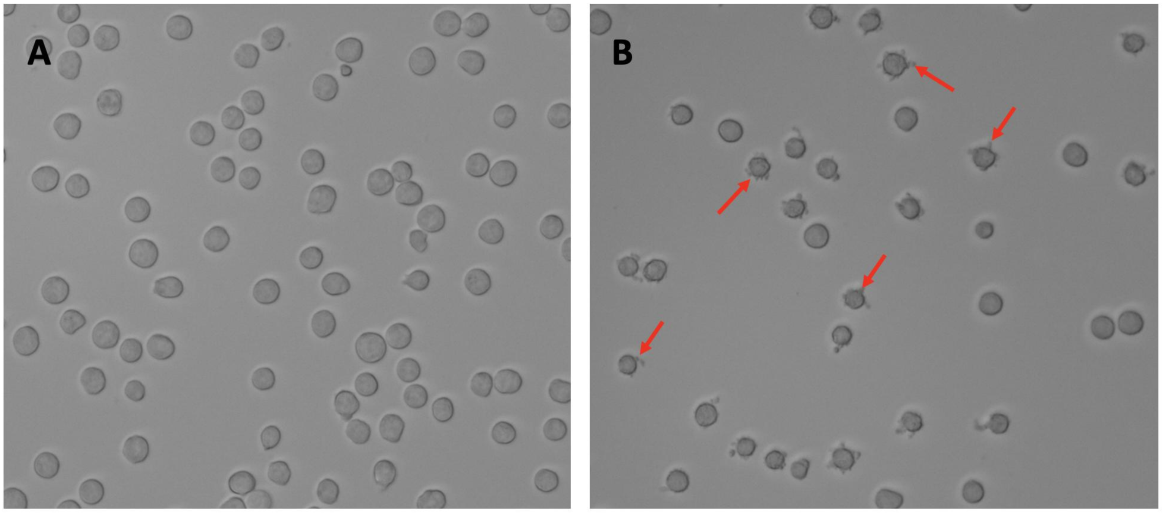

Figure S4. Microscope image of deposited PDA particles on the cell membrane, Related to Figure 2.

A) the control, B) PDA produced on the cell membrane as it is described in Supporting Information.

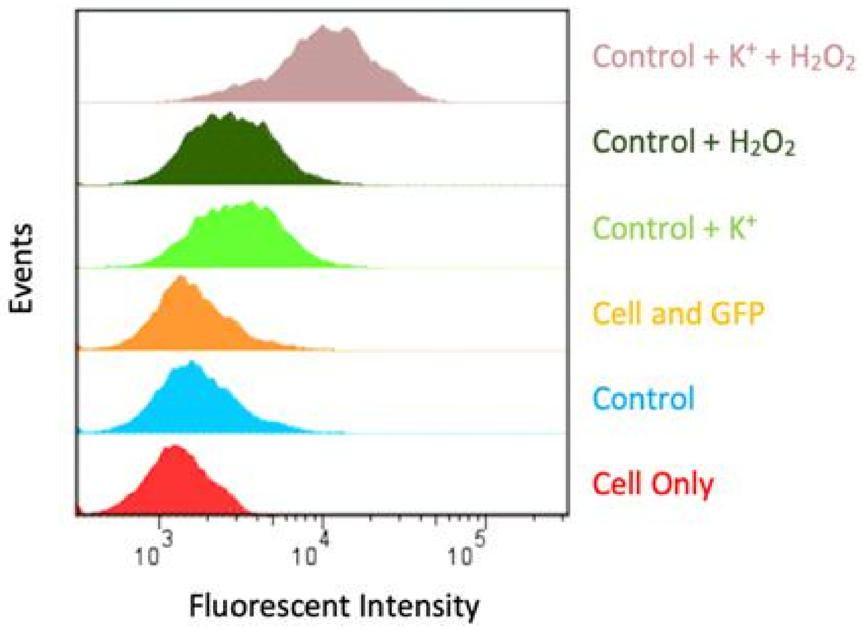

Figure S5. PDA Produced and Deposited on Cell Membrane, Related to Figure 2.

Flow cytometry histogram of CCRF-CEM cells treated with a low concentration of EGFP (100 nM), which leads to the low amount of attached EGFP to the cell membrane, as well as a smaller shift in flow cytometry histogram, rather than higher concentration of EGFP.

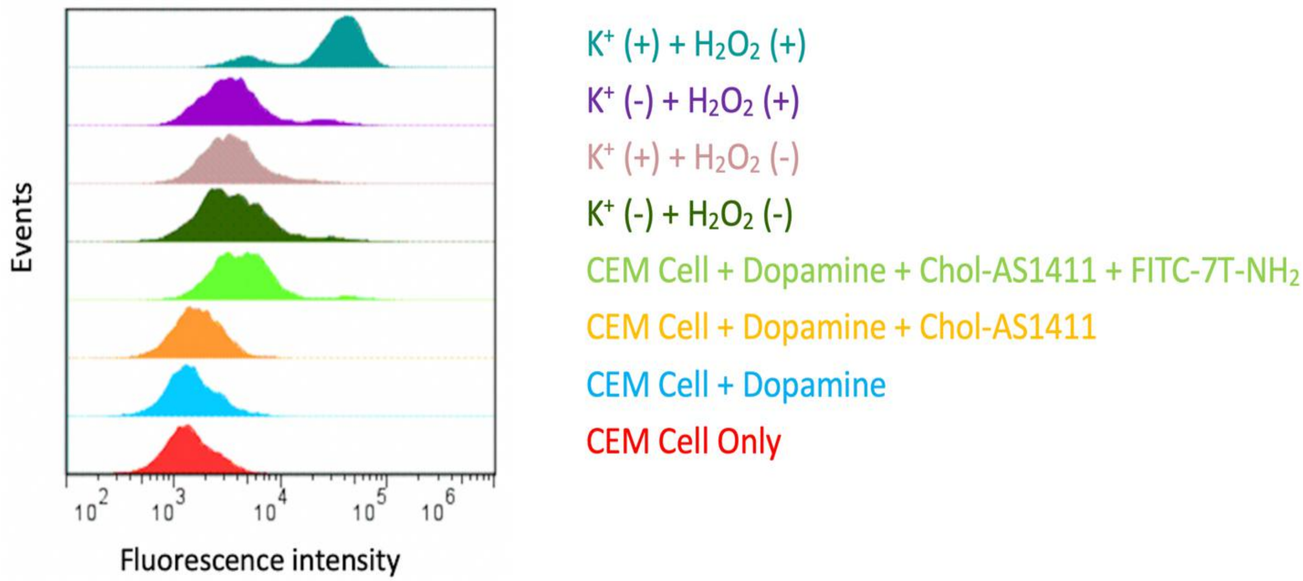

Figure S6. PDA Produced and Deposited on Cell Membrane, Related to Figure 2.

Flow cytometry histogram of CCRF-CEM cells treated with various reagents. FITC-7T-NH2, which is a short oligonucleotide sequence coupled directly to fluorescein isothiocyanate (FITC) and amino modifier, was used as the amino-containing detection molecule. Results show FITC-7T-NH2 attached to the deposited PDA on cell membrane, but only in the presence of both K^+ and H_2O_2 reagents, generating a good amount of fluorescence signal on the cell membrane and a shift in flow cytometry histogram.

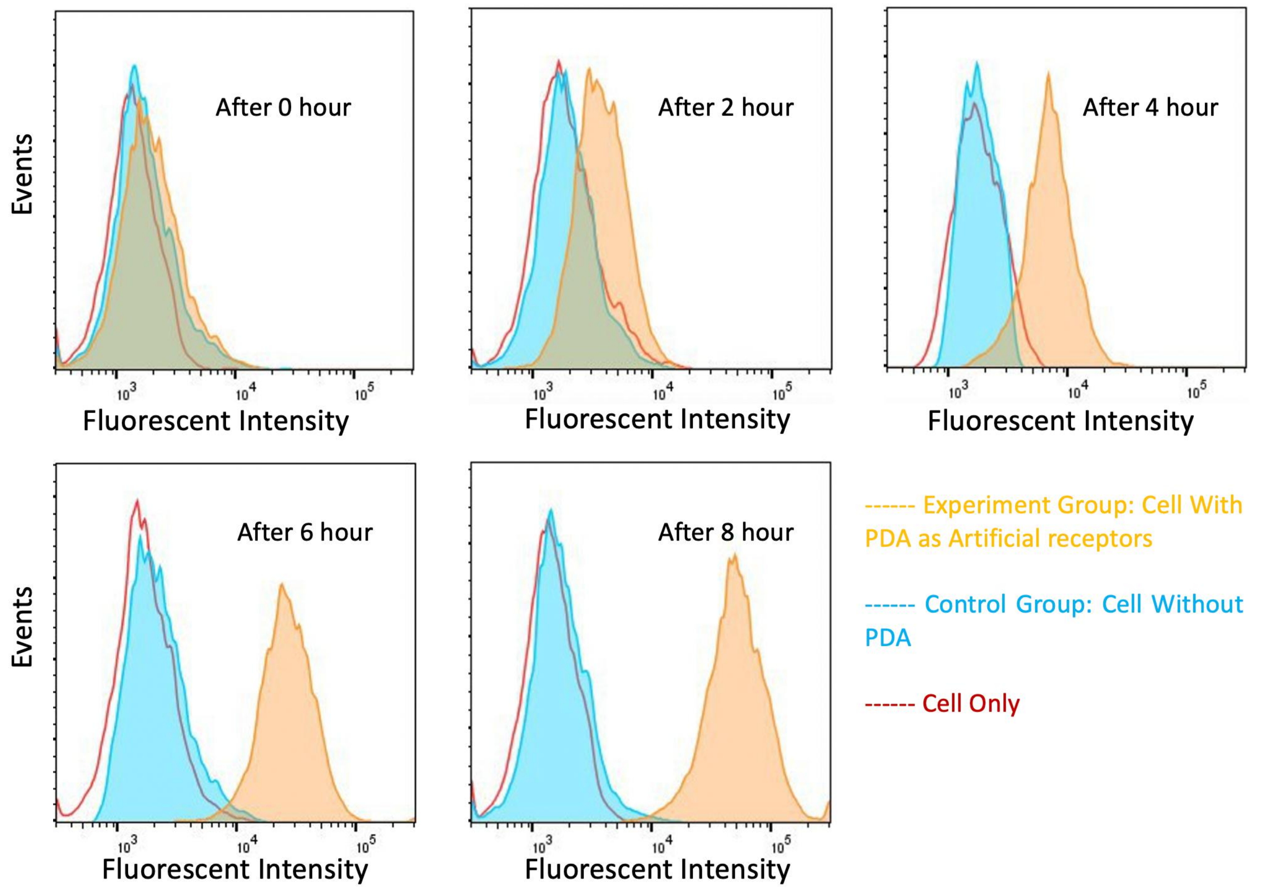

Figure S7. Cell uptake of EGFP as the protein model drug investigated using flow cytometry, Related to Figure 2.

Cell uptake of EGFP (50 nm) as the protein model drug investigated using flow cytometry. After PDA generated on the cell membrane, EGFP (50 nM) added as protein model drug owing to its fluorescence activity. Then the cells were incubated at 4 ºC for 1 hour and after 3 washing steps, the medium added to them and incubated at 37 ºC for 2h, 4h, 6h, and 8h. Before flow cytometry analysis, the cells were treated by trypsin for 3 minutes to remove all remaining EGFP on the cell membrane. Then after addition of medium and centrifugation, the cells were washed two times and trypsin was removed and finally flow cytometry analysis conducted to examine the cell uptake of the model drug.

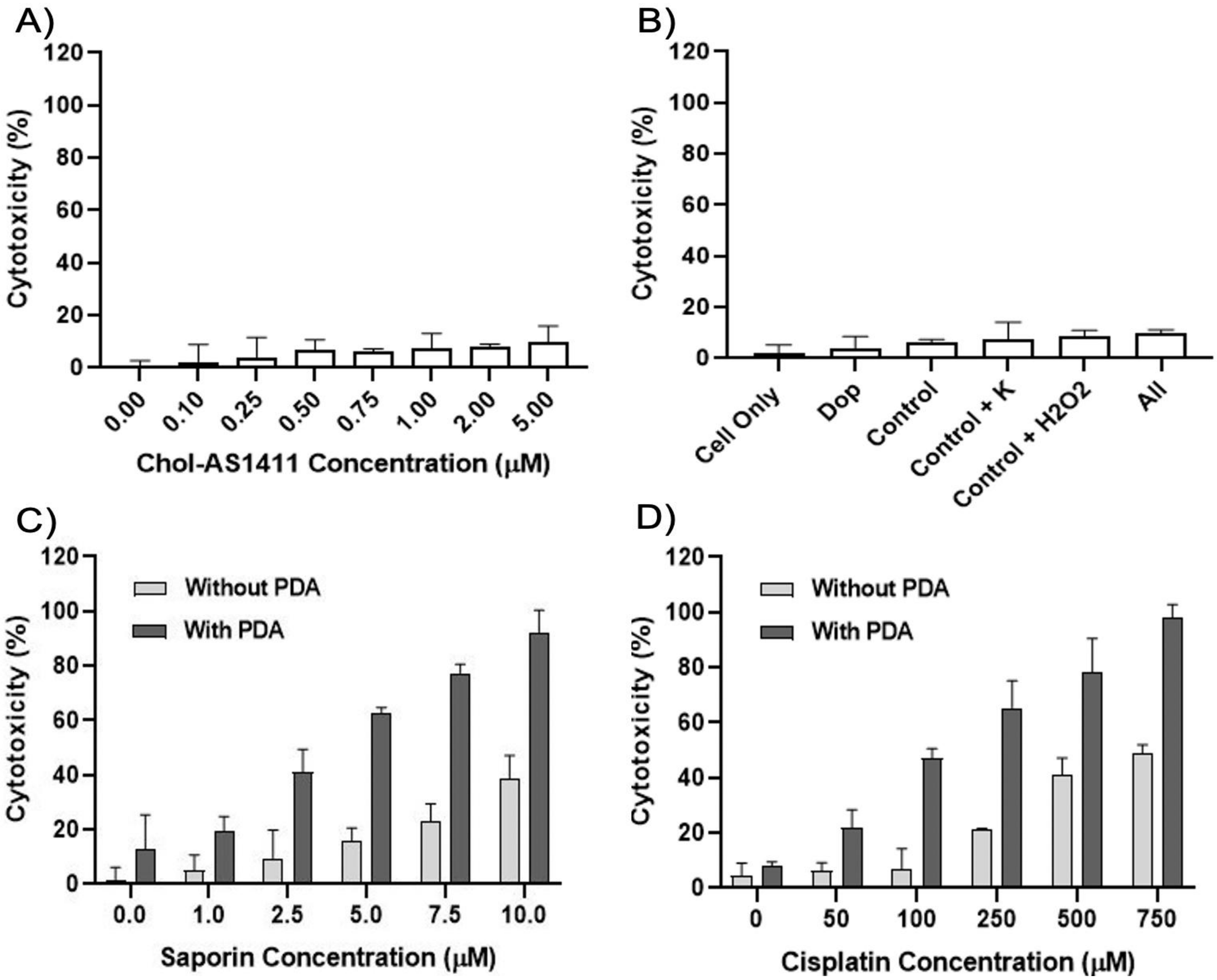

Figure S8. Cytotoxicity of CCRF-CEM cells treated with various reagents followed by verification of cytotoxicity by LDH toxicity assay, Related to Figure 3.

The error bars represent the standard deviation of three parallel experiments. A) Cells treated with different concentration of Chol-AS1411. B) Cells treated with PDA reagents, Group Dop: dopamine only; Group Control: dopamine and hemin-lipid-AS1411 without K+ and H2O2. C) Cells treated with different concentration of saporin (with/without artificial receptor). D) Cells treated with different concentration of cisplatin (with/without artificial receptor). The "Without PDA" labels in Figure 8C and 8D contains all the compounds just dopamine, and all the reaction condition kept exactly same with the "With PDA" wells.

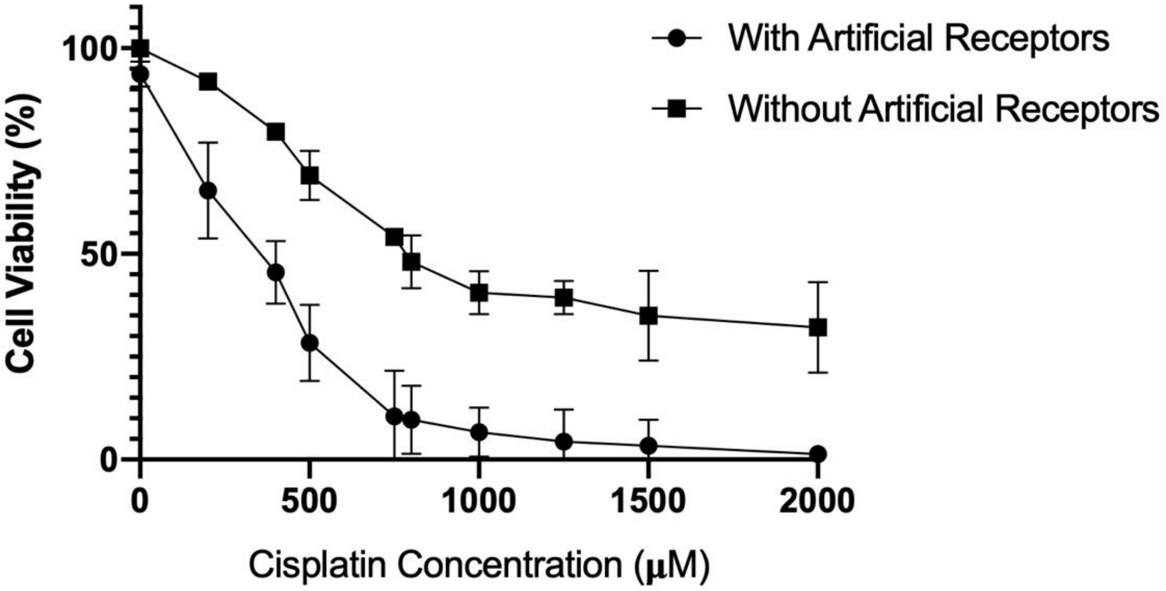

Figure S9. Cell viability of CCRF-CEM cells treated with different concentration of cisplatin, Related to Figure 3.

Cell viability of CCRF-CEM cells treated with different concentration of cisplatin (with/without artificial receptor), followed by verification of cell viability by MTS assay. The error bars represent the standard deviation of three parallel experiments.

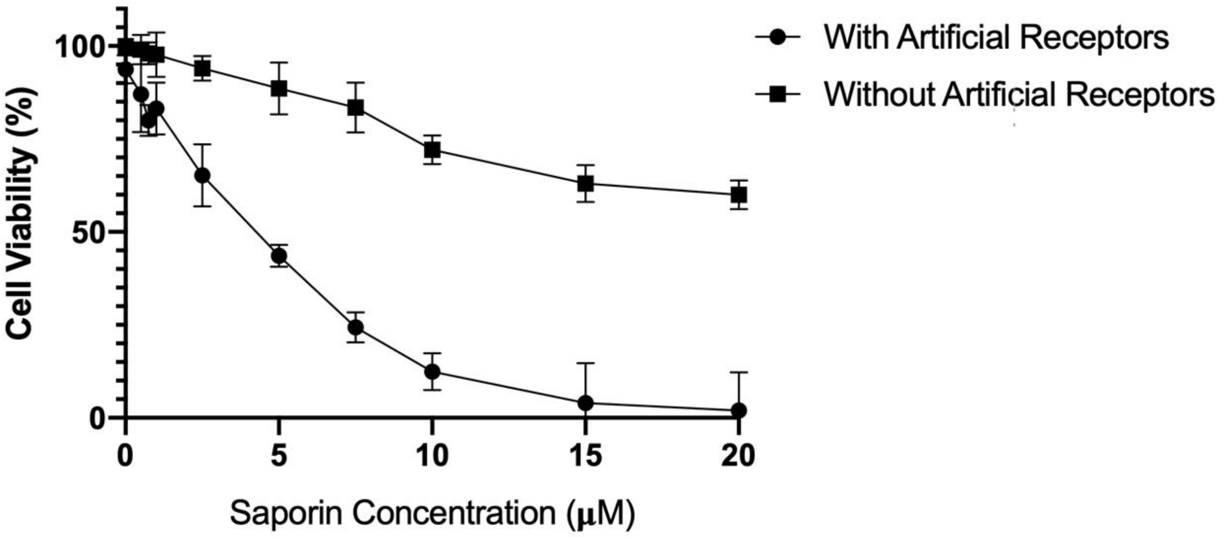

Figure S10. Cell viability of CCRF-CEM cells treated with different concentration of saporin, Related to Figure 3.

Cell viability of CCRF-CEM cells treated with different concentration of saporin (with/without artificial receptor), followed by verification of cell viability by MTS assay. The error bars represent the standard deviation of three parallel experiments.

Table S1. All oligonucleotide sequences used in this study, Related to Figure 1.

Name	Detailed Sequence Information
10T-AS1411	TTT TTT TTT TGG TGG TGG TGG TTG TGG TGG TGG TGG
Chol-AS1411	Cholesterol - TTT TTT TTT TGG TGG TGG TGG TTG TGG TGG TGG TGG
FITC-7T-NH_2	FITC - TTT TTT T - NH_2 - TTT TTT TTT TTT TT

Transparent Methods

1. Experimental Procedures

1.1 Materials

All used reagents were purchased from Thermo Fisher Scientific and Sigma-Aldrich unless otherwise stated. All DNA synthesis and modification reagents were purchased from Glen Research Corp. and ChemGenes Corp. DNA grade water from Fisher Scientific was used in all experiments, and phosphate-buffered saline (1X PBS) was purchased from Fisher Scientific. Also, 4-(2-Hydroxyethyl) piperazine-1-ethanesulfonic acid (HEPES buffer, 1M) was purchased from Thermo Fisher Scientific. The hemin stock solutions (5 μM) were made in dimethyl sulfoxide (DMSO) and stored in the dark and frozen at -20 °C.

1.2 Purification and Synthesis of Oligonucleotides

All oligonucleotides were synthesized with an ABI 3400 DNA synthesizer (Applied Biosystems, Inc., Foster City, CA) established on solid-state phosphoramidite chemistry at a 1 μmol scale. All synthesis started from controlled-pore glass (CPG) column and Cholesteryl-TEG phosphoramidite, and amino modifier coupled immediately onto the 3'-end of oligonucleotides. Deprotection procedure of all DNA sequences was carried out according to the instructions prepared by the manufacturer (Glen Research Corp.). Afterwards, by adding 2.5 times volume of cold ethanol and 1/10 volume of 3 M sodium chloride, DNA sequences were precipitated and placed in a -20°C freezer for 40 minutes. Later, by centrifuging for 20 minutes at 4,000 rpm, all precipitated DNA sequences were collected.

Purification of all deprotected DNA has been done by using reverse-phase high-performance liquid chromatography (HPLC) (ProStar, Varian, Walnut Creek, CA, USA) with a C18 column

(Econosil, 5μM, 250 X 4.6 mm). Acetonitrile and 0.1 M triethylamine acetate (TEAA) aqueous solution was used as the mobile phase in HPLC. After HPLC, all collected samples were vacuum dried, and sequences with DMT were treated with 80% acetate acid for 20 minutes at room temperature and finally precipitated by using 5/4 times volume of ice-cold ethanol and 1/10 volume of 3 M sodium chloride.

All sequence concentrations were quantified by measuring the absorbance at 260 nm using a UV-1800 UV spectrophotometer (Shimadzu Corp.).

1.3 Cell Culture

The CCRF-CEM (CCL-119 T-cell, human acute lymphoblastic leukemia), MDA-MB-231, and MCF10A cell lines was purchased from American Type Culture Collection (ATCC, Manassas, VA, USA). RPMI 1640 medium (Gibco®, Life Technologies, Carlsbad, CA) supplemented by 10% fetal bovine serum (FBS, heat inactivated) (Gibco®, Life Technologies, Carlsbad, CA) and 1% penicillin-streptomycin (PS) (Life Technologies, Carlsbad, CA) were used to culture CCRF-CEM cells in humidified atmosphere containing 5% CO_2 at 37 °C. DMEM medium (Gibco®, Life Technologies, Carlsbad, CA) supplemented by 10% fetal bovine serum (FBS, heat inactivated) (Gibco®, Life Technologies, Carlsbad, CA) and 1% penicillin-streptomycin (PS) (Life Technologies, Carlsbad, CA) were used to culture MDA-MB-231 and MCF10A cells in humidified atmosphere containing 5% CO_2 at 37 °C.

1.4 Polydopamine Production

After synthesis and purification of the 10T-AS1411 probe, a 40 μL solution (10 μM) was heated at 95 °C for 5 minutes and then cooled to room temperature gradually before use. Then K^+ ion

solution (40 μL, 200 mM) was added to the probe solution, and DNA sequences were allowed to fold into G-quadruplex conformation at room temperature for 40 minutes. Next, to produce hemin-G quadruplex DNAzyme, 20 μL of hemin solution (5 μM) were added to the mixture and incubated for 50 minutes at room temperature. Then hydrogen peroxide (H_2O_2) (40 μL, 2.5 mM) with final concentration of 500 μM was added to the constructed hemin-G quadruplex DNAzyme, and 2 minutes later, dopamine hydrochloride solution (60 μL, 100mM) was added to the mixture. Polydopamine was checked 30 minutes later by measuring absorbance at 450 nm with a microplate reader (CLARIOstar; BMG Labtech).

1.5 Transmission Electron Microscopy (TEM) Analysis

Ten microliters of sample were dropped on a carbon-coated copper grid for two minutes. Then a piece of filter paper was used to wick away extra solution, and the grid was air dried for TEM analysis. The grid was observed on a Hitachi H7000 microscope. The image was achieved with a slow-scan charge-coupled device (CCD) camera (Veleta 2k x 2k).

1.6 Flow Cytometry

Chol-AS1411 probe solution was heated at 95 °C for 5 minutes and then cooled to room temperature gradually before use. CCRF-CEM cells with a concentration of 2 X 10^5 cells/well were washed with ice-cold 50 mM HEPES buffer. After centrifugation at 1300 rpm for 3 minutes, cells were incubated with the chol-AS1411 solution in HEPES buffer (final concentration = 2 μM) at 4 °C for 40 minutes. Then K^+ ion solution, hemin, and H_2O_2 were added to the cells with the same procedure as that of the last step. After dopamine hydrochloride solution was added, the cells were incubated at 4 °C for 30 minutes. Then, cells were washed

twice by ice-cold 50 mM HEPES buffer to remove excess probes and polydopamine. In this step, the enhanced green fluorescent protein (EGFP) (100 nM or 500 nM) or FITC-NH_2 Probe (3 μM) was added to the cells to detect the production of polydopamine, and after 20 minutes, the cells were washed three times and resuspended in HEPES buffer for flow cytometry analysis.

EGFP and FITC fluorescence were measured by BD ™ Acuuri C6 flow cytometer, and 10,000 events were counted by setting EGFP and FITC channel under the same parameters. FlowJo software was used for data analysis.

1.7 Confocal Microscopy Study

Confocal laser scanning microscopy (CLSM) imaging was used to monitor the production of polydopamine. To ready cells for imaging, the procedure was the same as that for flow cytometry, but the concentration of EGFP increased to 2 μM. After adding EGFP and waiting for 20 minutes, the cells were washed three times and resuspended in 2% formalin solution to fix the cells for 7 minutes, and they were removed again, followed by the addition of 4′,6-diamidino-2-phenylindole (DAPI). After 10 minutes, the cells were washed for fluorescence imaging with a Leica TCS SP5 confocal microscope. All images were analyzed finally with LAS AF.

1.8 Cell Viability Study

MTS assay (3-(4,5-dimethylthiazol-2-yl)-5-(3-carboxymethoxyphenyl)-2-(4-sulfophenyl)-2H-tetrazolium)) (Promega, Madison, WI, USA) was used for all cell cytotoxicity experiments, and microplate reader (CLARIOstar, BMG Labtech) used to collect the absorbance of cells at 490 nm.

Cytotoxicity of chol-AS1411 was measured initially. CCRF-CEM cells (30,000) were washed and allocated to wells of a 96-well flat bottom, and 100 μL of cold 50 mM HEPES buffer with the desired concentration of cholesterol-AS1411 (from 0 μM to 5 μM) were added and incubated at 37 °C in 5% CO_2 for 3 hours. After removing the supernatant, the cells were washed three times with ice-cold 50 mM HEPES buffer. Then the cells were resuspended in RPMI-1640 complete medium (1% antibiotics (100 U/ml penicillin and 100 μg/ml streptomycin) and 10% Fetal Bovine Serum (FBS)) and incubated for 48 hours. After washing the cells, MTS reagent (20 μL) was added to each well with 100 μL of fresh medium and incubated for 1 hour. Next, the absorbance was collected and normalized to the absorbance of just fresh medium and MTS reagent. Concentrations < 5μM of chol-AS1411 are not toxic to CCRF-CEM cells.

The cytotoxicity of producing PDA was measured afterward. CCRF-CEM cells (30,000) were washed and allocated to wells of a 96-well flat bottom, and polydopamine was produced in vitro as discussed before. After dopamine hydrochloride solution was added, the cells were incubated at 4 °C for 30 minutes. Then, cells were washed by ice-cold 50 mM HEPES buffer. Next, they were resuspended in RPMI-1640 complete medium and incubated for 48 hours, followed by MTS assay as the last step. The production of polydopamine by using hemin-G quadruplex DNAzyme was not cytotoxic to CCRF-CEM cells.

To investigate the potency of the artificial receptor for targeted drug delivery, 30,000 CCRF-CEM cells were washed and allocated to wells of a 96-well flat bottom, and polydopamine was produced in vitro as discussed before. After dopamine hydrochloride solution was added, the cells were incubated at 4 °C for 30 minutes. Then, cells were washed by ice-cold 50 mM HEPES buffer and gradient concentration of saporin (0 to 10 μM) and cisplatin (0 to 750 μM) in RPMI-1640 complete medium was added to the cell solution and then incubated at 37 °C in 5% CO2

for 6 hours. Cells were then washed, and fresh RPMI-1640 complete medium added again to the cell solution, followed by at 37 °C in 5% CO2 for 48 hours. After washing the cells, MTS reagent (20 μL) was added to each well with 100 μL of fresh medium and incubated for 1 hour. Next, the absorbance was collected and normalized to the absorbance of just fresh medium and MTS reagent. All steps were performed on cells in the same manner without the artificial receptor. However, the reagents to produce PDA were not added. The IC50 values were calculated as the concentrations reducing proliferation of cells by 50% and are given as the means and errors of 3 independent experiments (Figure S3).

1.9 Cytotoxicity Study

The cytotoxicity effect of PDA production via LDH cytotoxicity assay was investigated. In this cytotoxicity assay, the cell death measured by cell-free supernatants from cells in culture and then extra absorption of PDA could not affect its result. Cell death was evaluated via measuring the release of cytoplasmic Lactate Dehydrogenase (LDH) isoenzymes from damaged cells using the LDH toxicity assay kit, according to the manufacturer's instructions (Thermo Fisher Scientific). Briefly, the cells treated exactly same as it described in “Cell Viability Study” section above, but instead of MTS cytotoxicity assay, LDH cytotoxicity assay was used. 50μL of each sample medium was transferred to a 96-well flat bottom plate and 50μL of LDH reaction mixture was added to each well. After 30 minutes incubation at room temperature, 50 μL of stop solution added to each well. The final absorbance was measured at 490 nm and all experiments were conducted in triplicate.
